# Editorial: Multi-omics in cardiomyopathies causing heart failure: from mechanism to treatment

**DOI:** 10.3389/fcvm.2024.1405813

**Published:** 2024-04-17

**Authors:** Chen Yao

**Affiliations:** Department of Informatics and Predictive Sciences, Bristol Myers Squibb, New York, NY, United States

**Keywords:** cardiomyopathies, multi-omics, precision medicine, transcriptomics, proteomics, biomarkers

**Editorial on the Research Topic** Multi-omics in cardiomyopathies causing heart failure: from mechanism to treatment

In the realm of cardiovascular diseases, cardiomyopathies stand as formidable adversaries, presenting multifaceted challenges to clinicians and researchers. Among these, dilated cardiomyopathy (DCM) and hypertrophic cardiomyopathy (HCM) loom large, each with its distinct pathophysiological intricacies and diagnostic conundrums. Recent breakthroughs in integrated bioinformatics, experimental validation, and precision medicine have cast a promising light on the landscape of these conditions, offering novel insights into their molecular underpinnings and diagnostic potential.

The study by Zhang et al. delves into the complex terrain of DCM, a condition characterized by ventricular dilation and impaired systolic function, with elusive etiology. Leveraging the power of bioinformatic algorithms, the researchers embarked on a comprehensive journey to identify robust biomarkers and unravel the intricate landscape of immune cell infiltration in DCM myocardial tissues. Through meticulous analysis, they unearthed key immune cells, such as activated NK cells and Eosinophils, alongside hub genes like ASPN, CD163, IL10, and LUM, shedding light on potential diagnostic avenues and underlying mechanisms driving DCM progression.

In a parallel endeavor, Lander et al. embarked on a quest to decipher the enigma of late gadolinium enhancement (LGE) on cardiac magnetic resonance imaging (CMR) in the HCM population. Recognizing LGE as a harbinger of myocardial fibrosis and heightened risk of sudden cardiac death, the researchers pioneered the use of plasma proteomics profiling to discern patients with and without LGE. Their findings underscored the discriminative prowess of plasma proteomics, offering a tantalizing glimpse into the realm of personalized medicine and refined risk stratification in HCM.

Meanwhile, Zheng et al. set their sights on constructing a genetic diagnosis model for DCM, harnessing the power of weighted gene co-expression network analysis (WGCNA) to unveil key genes and pathways underpinning DCM pathogenesis. Their innovative approach culminated in the development of a LASSO diagnostic model boasting exceptional accuracy across diverse cohorts, heralding a new era of precision medicine in the diagnosis and management of DCM.

On a molecular level, Chauhan and Sowdhamini delved into the intricate transcriptomic landscape of primary cardiomyopathies, unveiling perturbations in arachidonic acid metabolism as a common thread linking diverse cardiomyopathic phenotypes. Their findings illuminate the intricate interplay between metabolic dysregulation and cardiac pathology, paving the way for targeted therapeutic interventions and precision medicine approaches.

In a convergence of multi-omics integration, Portokallidou et al. undertook a comprehensive analysis to delineate the genetic expression and protein signature of both DCM and ischemic cardiomyopathy (ICM). Their meticulous bioinformatics approach unearthed shared and distinct molecular pathways, offering invaluable insights into the molecular underpinnings of HF subphenotypes and paving the way for the development of novel diagnostic biomarkers and therapeutic targets.

Lastly, Nomura and Ono championed the cause of precision and genomic medicine in the realm of cardiomyopathies, advocating for a holistic approach that integrates genetic testing, single-cell analysis, and genome-wide research to unravel the intricate tapestry of cardiac pathology. Their visionary outlook heralds a new dawn in cardiomyopathy research, where personalized interventions and targeted therapies hold the promise of transforming clinical outcomes and improving patient care.

Collectively, these pioneering studies epitomize the relentless pursuit of knowledge and innovation in the field of cardiomyopathy research. As we stand at the cusp of a new era in cardiovascular medicine, fueled by cutting-edge technologies and interdisciplinary collaboration, the horizon brims with promise and potential. Armed with insights gleaned from integrated bioinformatic algorithms, experimental validation, and precision medicine approaches, we march steadfastly towards a future where the scourge of cardiomyopathies is met with resilience, resolve, and unwavering scientific ingenuity.

